# Imunne system and schyzophrenia

**DOI:** 10.1192/j.eurpsy.2022.1806

**Published:** 2022-09-01

**Authors:** A. Almeida, T. Teixeira, J. Quarenta

**Affiliations:** 1 Centro Hospitalar do Tâmega e Sousa, Departamento De Psiquiatria E Saúde Mental, Guilhufe-Penafiel , Portugal; 2 Centro Hospitalar do Tâmega e Sousa, Departamento De Psiquiatria E Saúde Mental, Penafiel, Portugal

**Keywords:** neuroinflammation, inflammation, schizophrénia, Immune system

## Abstract

**Introduction:**

Schizophrenia affects approximately 1% of the world population, having a devastating impact not only in patients but in all society. As a result, it has been subject of extensive investigation and the presence of certain genes was associated with an increased risk of developing schizophrenia. However, the presence of these genes is not sufficient, therefore, other factors are necessarily involved.Observation of the association between schizophrenia and inflammatory states of the Central Nervous System led to the hypothesis that a dysfunction of the immune system may play a central role in this process.

**Objectives:**

In this work we intend to make a brief review of the existing literature related to the immunological theory of schizophrenia.

**Methods:**

A bibliographic research was conducted in Medline library using the following terms: “schizophrenia and immune system”; “schizophrenia and inflammation” and “schizophrenia and neuroinflammation”.

**Results:**

The survey results reveal increasing evidence of the key role of the immune system in schizophrenia. Several studies show benefits of treatment with anti-inflammatory drugs in patients at an early stage of the disease. In the same way, it was verified that pro and anti-inflammatory cytokines influence glutamatergic transmission and tryptophan metabolism. Furthermore, the decrease in microglial activity appears to have a beneficial effect on schizophrenia.

**Conclusions:**

Future will say if neuroimmunology mechanisms are primary or a secondary consequence in Schizophrenia. Recent discoveries in this area are encouraging and open the possibility of new therapeutic targets and new therapeutic approaches to this disease.

**Disclosure:**

No significant relationships.

